# Correction: Microglia-secreted TNF-α affects differentiation efficiency and viability of pluripotent stem cell-derived human dopaminergic precursors

**DOI:** 10.1371/journal.pone.0318359

**Published:** 2025-01-24

**Authors:** The PLOS ONE Staff

## Notice of Republication

This article was republished on January 14, 2025, to correct errors in the affiliations, in Figure 2, and in the captions for Figures 2 and S1, that were introduced during the typesetting process. The publisher apologizes for these errors. Please download this article again to view the correct version. The originally published, uncorrected article and the republished, corrected articles are provided here for reference.

## Supporting information

S1 File Originally published, uncorrected article. (PDF)

S2 File Republished, corrected article. (PDF)

## References

[1] Wenker SD, Farias MI, Gradaschi V, Garcia C, Beauquis J, Leal MC, et al. (2023) Microglia-secreted TNF-α affects differentiation efficiency and viability of pluripotent stem cell-derived human dopaminergic precursors. PLoS ONE 18(9): e0263021. 10.1371/journal.pone.0263021 37751438 PMC10521980

