# Supplementary figures and images for: Microglia-secreted TNF-α affects differentiation efficiency and viability of pluripotent stem cell-derived human dopaminergic precursors
Source: PLoS One. 2023 Sep 26;18(9):e0263021. doi: 10.1371/journal.pone.0263021 (PMC10521980; doi:10.1371/journal.pone.0263021)

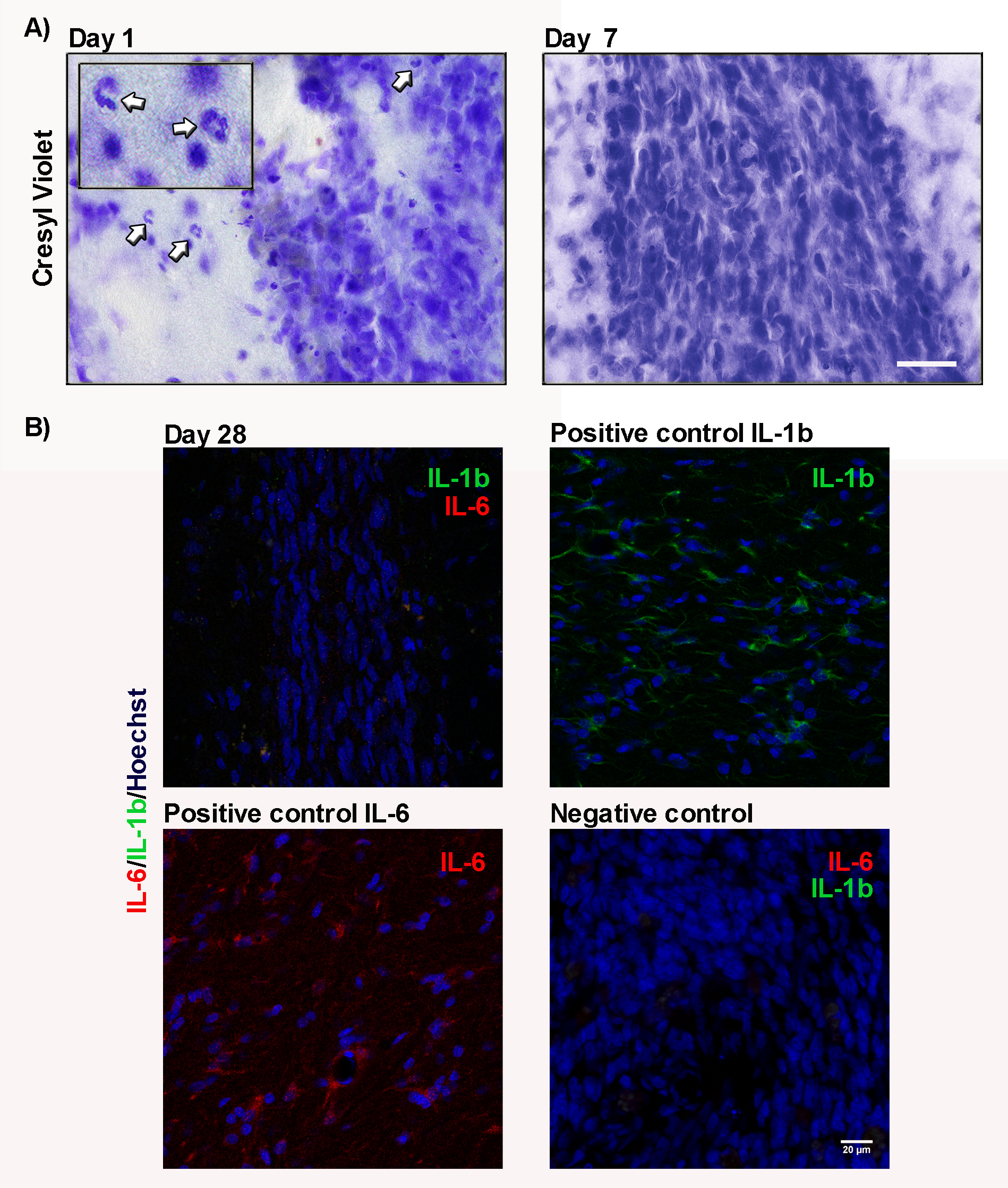

Supplement: S1 Fig — (TIF) [file pone.0263021.s001.tif]
